# Quality-Adjusted Life-Years Outcome 1 Year After Surgery by Robotics-Assisted Sacral Hystero-Colpopexy Versus Vaginal Mesh for Pelvic Organ Prolapse Repair

**DOI:** 10.1007/s00192-025-06242-7

**Published:** 2025-08-13

**Authors:** Georgios Poutakidis, Kirk Geale, Edward Morcos

**Affiliations:** 1 Department of Clinical Sciences, Division of Obstetrics and Gynecology, Karolinska Institutet Danderyd Hospital, 176 77 Stockholm, Sweden; 2 Department of Obstetrics and Gynecology, Danderyd University Hospital, 182 88, Danderyd, Stockholm, Sweden; 3 Quantify Research, 112 21 Stockholm, Sweden

**Keywords:** Apical prolapse surgery, Robotics-assisted sacral hystero-colpopexy, Uphold™ vaginal mesh, QALY, HR-QoL, 15D, EQ-5D-3L

## Abstract

**Introduction and Hypothesis:**

The aim of this study was to compare the quality-adjusted life-years (QALYs) attained 1 year after robotics-assisted sacral hystero-colpopexy (RASC) versus Uphold™ vaginal mesh surgery for pelvic organ prolapse repair.

**Methods:**

This was a secondary analysis of a previously published cohort study. A total of 65 patients who underwent RASC and 71 who underwent the Uphold™ procedure completed the 15-dimensional (15D) and the EuroQol five-dimensional three-level (EQ-5D-3L) instruments measuring health-related quality of life (HR-QoL). All patients had symptomatic and anatomical apical prolapse (POP-Q C ≥ stage II) with or without other vaginal wall defects. Changes in HR-QoL instruments were calculated and compared with minimal important change (MIC) thresholds and QALYs gained were estimated for each intervention.

**Results:**

The 15D and EQ-5D-3L mean index scores were improved from preoperatively to 1 year after RASC ([0.88 ± 0.10 to 0.90 ± 0.01] and [0.85 ± 0.1 to 0.90 ± 0.1]) and after Uphold™= ([0.87 ± 0.1 to 0.89 ± 0.1] and [0.86 ± 0.1 to 0.93 ± 0.1], *p* 0.024 to *p* < 0.001) with no significant difference between cohorts. Prolapse-related 15D profile index measures, including discomfort, sexual activity, and distress were significantly improved after RASC (*p* = 0.039 to < 0.001), whereas excretion, discomfort, and sexual activity were significantly improved after the Uphold™ (*p* = 0.009 to < 0.001). The improvement in 15D scores from baseline to 1-year follow-up of + 0.026 for RASC and + 0.025 for Uphold™ exceeded the MIC, indicating meaningful improvements in patient quality of life. The overall 1-year QALY gain was estimated to be 0.90 ± 0.1 in the RASC and 0.88 ± 0.1 in the Uphold™ cohorts (*p* < 0.001), with no significant difference between the two interventions (*p* = 0.514).

**Conclusions:**

The RASC and Uphold™ are both meaningful surgical treatments for prolapse, with significant improvement in the HR-QoL and the 1-year QALY gain and with no significant difference between the two surgeries.

## Introduction

Sacrocolpopexy (SCP) using polypropylene mesh implant is an efficacious surgery for apical prolapse [1]. The method may be used for apical support of the uterus (hysteropexy) or the vaginal vault if previous hysterectomy was performed [1–3]. SCP may be performed by abdominal (ASC), laparoscopic (LSC) and robotics-assisted (RASC) surgery techniques [1–3]. The use of minimally invasive surgery by the LSC and RASC is growing faster in order to further lower the morbidity and improve the clinical outcomes [4–6]. The Uphold™ Vaginal polypropylene mesh Support System for pelvic organ prolapse surgical repair was reported to be associated with significantly improved subjective and anatomical outcomes at the short- and long-term follow-ups [7–11]. The SCP was previously considered the standard surgery for apical prolapse [1]. However, recent studies indicated comparable clinical outcomes between the RASC and the Uphold™ [12, 13]. The Uphold™ mesh was withdrawn from the market in 2019 following the FDA decision to ban transvaginal mesh [14]. However, ongoing studies to evaluate the outcomes after the vaginal mesh surgeries in the long term are encouraged by the medical authorities [14, 15].

Generic health-related quality of life (HR-QoL), as estimated by the 15-dimensional (15D) instrument, has been shown to be improved 1 year after surgery using the Uphold™ [16]. The improvement in HR-QoL after surgery using the Uphold™ correlated with improvement in the prolapse-related 15D profile index scores [16]. Two common generic (i.e., not disease specific) quality-of-life instruments include the 15D and the EuroQol five-dimensional three-level (EQ-5D-3L) instruments [17, 18]. The quality-adjusted life-years (QALYs) is a two-dimensional outcome measured by weighing the length of life (survival) by quality of life, measured by instruments such as the 15D or EQ-5D-3L [19]. There is a lack of knowledge when comparing how surgeries with the RASC and the Uphold™ may affect the generic HR-QoL and the QALY gain. Therefore, the aim of the present study was to compare the 1-year change in HR-QoL and QALY gains between the RASC and Uphold™ mesh.

## Materials and Methods

The primary outcome of the present study was to compare the 1-year QALYs between the RASC and the Uphold™ mesh surgeries, whereas the secondary outcome was to compare changes in HR-QoL outcome between the two surgeries. This was a secondary analysis of data prospectively collected from a previously published cohort study of women undergoing abdominal versus vaginal mesh repair [13]

### Patients and Surgery

The surgeries were performed during 2018–2019 at three clinics in Stockholm, and Västerås, Sweden, as previously described in detail [13]. A total of 72 patients operated on using RASC and 73 patients operated on using the Uphold™ were included in the study. All operated patients suffered symptomatic apical prolapse (uterine or vaginal vault, POP-Q C stage ≥ 2) with or without other concomitant vaginal compartment prolapse. Choice of surgical method, i.e., the RASC or the Uphold™, is dependent on the surgeon. However, only postmenopausal women were included in the Uphold™ cohort. The exclusion criteria included current or previously treated pelvic organ cancer; cervical elongation (more than 3 cm); severe rheumatic disease and connective tissue disorder [13].

The RASC surgery procedure was performed following an established technique, as previously described by three experienced robotic surgeons using the Da Vinci surgical system (Intuitive Surgical, Sunnyvale, CA, USA) [13, 20]. For each individual patient, the Artisyn® Y-Shaped Mesh (Ethicon) or the Restorelle® XL Mesh (Coloplast) was adjusted by the operating surgeon to support the apical and vaginal wall defects [13, 20]. The peritoneum of the sacral promontory was exposed, and the pelvic sidewall was dissected, as well as the anterior vaginal wall and the bladder to the trigone area in order to be able to fix the anterior part of the mesh to the anterior vaginal wall. After dissection, the posterior mesh was then fixed to the posterior vaginal wall. The apical vaginal part of the mesh was fixed to the posterior wall of the cervix for hysteropexy, to the cervix stump in the case of a previous or at the time subtotal hysterectomy (supra-cervical hysterectomy), or to the vaginal vault (vaginal sacrocolpopexy) if women had had a previous total hysterectomy. The mesh was then passed through the upper part of the cardinal ligament, around the cervix on each side, to be further fixed down to the anterior vaginal wall at a maximum length of 4 cm depending on the size of the anterior vaginal wall prolapse size. The mesh was placed down on the anterior longitudinal ligament at the sacral promontory, medial to the right ureter, avoiding the hypogastric nerve [13, 20]. Nonresorbable stitches were used for mesh fixation. Finally, the peritoneum was closed with resorbable running sutures to cover the mesh.

The Uphold™ Vaginal Support System was used in a standardized surgical procedure in all patients and as previously described [13]. The two-armed Uphold™ mesh is a monofilament, microporous, and uncoated polypropylene mesh. Through a midline incision in the anterior vaginal wall, the Uphold™ vaginal mesh was fixed to the apical vaginal compartment (uterus, cervix stump, or vaginal vault). Using the Capio™ SLIM Suture Capturing Device (Boston Scientific), the two arms of Uphold™ mesh were passed through the sacrospinous ligament on both sides in order to suspend the apical vaginal part to the sacrospinous ligament level [7, 11, 13].

Concomitant prolapse surgery using native tissue repair included anterior and posterior colporrhaphies with, or without, perineorrhaphy if symptomatic prolapse existed. Single-dose prophylactic antibiotics were given to all patients in the two cohorts. None of the patients in the two cohorts received a midurethral sling procedure for stress urinary incontinence at the time of primary surgery in the RASC or Uphold™ cohorts.

### Anatomical Quantification by the Pelvic Organ Prolapse Quantification

The Pelvic Organ Prolapse Quantification (POP-Q) system was used for anatomical estimation of prolapse. POP-Q C indicates the apical vaginal segment state whereas POP-Q Ba and POP-Q Bp for the anterior and posterior vaginal walls, respectively. The POP-Q TVL assessed the total vaginal length and the POP-Q Pb for the perineal body [21]. All operated women had at least symptomatic apical prolapse (POP-Q stage ≥ 2) with or without other symptomatic vaginal compartment prolapse. The POP-Q optimal anatomical outcome after surgery was considered POP-Q stage 0 or 1 in the apical vaginal segment and the primary outcome measure.

### Demographics

Medical, socioeconomic, and surgery characteristics at baseline were collected. The Visual Analog Scale (VAS) was used for estimation of pain [22]. The VAS is a horizontal 11-point scale, where 0 indicates no pain and 10 points indicate severe pain. Surgery characteristics including anesthesia type, operating time, and hospital stay were also collected.

### 15-Dimensional, EuroQol Five-Dimensional Three-Level, and Health-Related Quality-of-Life Instruments

The HR-QoL was estimated using the 15D and the EuroQol EQ-5D-3L instruments with the UK Dolan tariffs [23, 24]. The 15D and EQ-5D-3L instruments were collected preoperatively, at hospital discharge, and 2 weeks, 3 months, 6 months, 9 months, and 1 year after the surgery.

The 15D includes 15 dimensions (profile index scores): mobility, vision, hearing, breathing, sleeping, eating, speech (communication), excretion, usual activity, mental function, discomfort, depression, distress, vitality, and sexual functions [16, 23]. Each dimension has five descriptive levels to choose from. The 15D can be used as a profile and a single index score measure. The 15D single index score is used to measure health-related quality of life. The 15D profile index scores of mobility, elimination (excretion), discomfort, distress, and sexual activity were considered prolapse-related symptoms. The valuation system is based on the application of multi-attribute utility theory. The single index or utility score represents the generic health-related quality of life on a maximum value of 1 (full health) to a lower bound of 0 (being dead). In addition to the utility values, the 15D also reports scores separately for each individual dimension in order to identify where the changes in quality of life take place [16, 23]. The generic minimal important change (MIC) of ±0.015 in the 15D single index score, if statistically significant, was considered a meaningful threshold for improvement or deterioration in health-related quality of life [25].

The EQ-5D-3L was also used to estimate the quality of life [24]. The instrument’s assessment dimensions include mobility, self-care, usual activities, pain/discomfort, and anxiety/depression.

Quality-Adjusted Life-Years were evaluated using the estimated 15D and EQ-5D-3L weights between the preoperative baseline visit to 1 year after surgery [18]. QALYs were calculated using the area under the curve approach. The average utility across each of two subsequent follow-up visits was multiplied by the duration of time between the two visits. Each of these utility-weighted time intervals was added to calculate the total QALYs gained in each treatment arm.

### Statistical Analysis

To compare the independent variables, we used the two-sided independent sample *t* test. The dependent *t* test (paired-samples *t* test) was used to compare the means of two related groups on the same continuous, dependent variable. For analysis of the statistical difference between nominal data we used the Chi-squared test of independence. Fisher´s exact test was used for analysis of the categorical data when classifying results in two different ways in order to examine the significance of the association, i.e., the contingency between the two kinds of classification. McNemar's test was used for analysis of the paired nominal data. Repeated measures ANOVA was used to test total values and to compare continued variables and differences in changes over time in follow-up between the two surgery cohorts. The Bonferroni post hoc test was used to limit the apparent statistical significance when analyzing the effect of multiple comparison follow-ups on the dependent variable. Repeated measures ANOVA was used to evaluate differences over time within groups and to compare between the groups. We used a multiple linear regression and stratified analysis of covariance (ANCOVA), as a general linear model that blends ANOVA and regression. This was in order to investigate whether patient’s socioeconomic, medical, and surgery characteristics were predictive of improvement in the estimated 15D, MIC, and QALYs. All missing data were considered missing without imputation of data, i.e., we used per-protocol statistical analysis. A post hoc power analysis concluded that a minimum of 67 individuals in each cohort (total 67*2 = 134) yielded 80% power to detect an association of the patient’s socioeconomic, medical, and surgery characteristics with the HR-QoL instrument when we use a linear regression model (two predictors) with an Cohen effect of f = 0.39 (medium effect). Statistical analysis was performed using the predictive analysis software (version 28, 2023; IBM@SPSS© Statistics, Chicago, IL, USA).

## Results

Preoperatively, 65 (90.3%) and 71 (97.3%) patients responded to the 15D questionnaire, whereas 61 (85%) and 63 (86.3%) patients responded at the 1-year follow-up in the RASC and the Uphold™ cohorts respectively. For the EQ-5D-3L questionnaire, 65 (90.3%) and 69 (94.5%) patients responded preoperatively, and 61 (85%) and 62 (85%) patients responded at the 1-year follow-up in the RASC and the Uphold™ cohorts respectively. The total number of available data is presented for each measurement in Tables 1, 2, 3, 4, and 5.

**Table 1:** Demographic, medical, socioeconomic, and surgery characteristics for the robotics-assisted sacral hystero-colpopexy (RASC) and Uphold™ groups

	RASC		Uphold™		
	Mean ± SD, CI, *n* (%)	Total	Mean ± SD, CI, *n* (%)	Total	*p* value
Age, years	58.8 ± 13.3 (55.6–62.1)	65	69.5 ± 8 (67.6–71.4)	71	< 0.001*
BMI (kg/m^2^)	25.3 ± 3.8 (24.3–26.2)	65	25.6 ± 3.1 (24.8–26.3)	71	0.585
Vaginal deliveries	2.3 ± 1 (2–2.5)	61	2.7 ± 0.9 (2.5–2.9)	68	0.023**
Parity	2.3 ± 1	61	2.7 ± 1	70	0.172
Caesarean section	0.2 ± 0.5 (0–0.3)	61	0.1 ± 0.4 (0–0.2)	68	0.339
Menopause vs still having menstruation	40 (64.5%)vs 22 (35.5%)	62	70 (98.6%) vs 1 (1.4%)	71	< 0.001*
Somatic diseases (none, CVS, other diseases)	24 (36.9%), 20 (30.8%), 21 (32.3%)	65	10 (14.1%), 38 (53.5%), 23 (32.4%)	71	0.004***
Physical training (0, 1–2, 3–4 times/week)	12 (19.7%), 24 (39.3%), 25 (41%)	61	21 (29.6%), 29 (40.8%), 21 (29.6%)	71	0.282
Job (retired vs working)	23 (35.4%) vs 42 (64.6%)	65	46 (64.8%) vs 25 (35.2%)	71	0.001*
Education level (elementary, upper secondary school, university education 3–6 years)	11 (17.7%), 20 (32.3%), 31 (50%)	62	20 (28.6%), 12 (17.1%), 38 (54.3%)	70	0.088
Annual income (< US$29,999, ≥ US$30,000 a year)	27 (45%), 33 (55%)	60	34 (52.3%), 31 (47.7%)	65	0.414
Hysterectomy prior to surgery vs still having a uterus	16 (24.6%), 49 (75.4%)	65	15 (21.1%), 56 (78.9%)	71	0.628
Primary vs recurrent prolapse surgery	44 (71%) vs 18 (29%)	62	32 (46.4%) vs 37 (63.6%)	69	0.004***
Previous surgery with mid-urethral sling vs none	5 (8.1%) vs 57 (91.9%)	62	7 (10.3%) vs 61 (89.7%)	68	0.661
Previous pelvic floor surgery (0, 1, ≥ 2 operations)	33 (53.2%), 21 (33.9%), 8 (12.9%)	62	19 (27.9%), 40 (58.8%), 9 (13.3%)	68	0.023**
Pain (VAS 0–10)	2.9 ± 2.4	64	3.1 ± 2.4	68	0.419
Anesthesia type (general, spinal)	65 (100%), 0 (0%)	65	7 (9.9%), 64 (90.1%)	71	< 0.001*
Operating time (min)	151.4 ± 38.7	65	67.5 ± 25.4	71	< 0.001*
Hospital stay, days	1.1 ± 0.3	65	1.2 ± 0.7	71	0.368

Two-sided independent sample *t* test compare between the independent variables

Chi-squared test of independence was used for comparative analysis of the nominal data and Fisher´s exact test for comparative analysis of the categorical data

*BMI* body mass index, *CI* confidence interval, *SD* standard deviation

*p* value < 0.05 was considered significant

*p value < 0.001

**p value < 0.05

***p value < 0.01

**Table 2:** The estimated 15-dimensional (15D) single index and sub-score profile index values

	Preoperative			6-months			1-year			*p-values *(preoperative, 6-months, 1-year)		
	RASC	Uphold™		RASC	Uphold™		RASC	Uphold™		RASC	Uphold™	RASC vs Uphold™
	Mean ± SD	Mean ± SD	*p–value*	Mean ± SD	Mean ± SD	*p–value*	Mean ± SD	Mean ± SD	*p–value*	*p*–value	*p*–value	
*N*	65	71		63	62		61	63		61	63	65 vs. 71
Mobility	0.98 ± 0.08	0.89 ± 0.16	< 0.001*	0.97 ± 0.12	0.94 ± 0.14	0.211	0.97 ± 0.09	0.92 ± 0.14	0.049***	0.553	0.081	0.090
Vision	0.98 ± 0.06	0.96 ± 0.09	0.261	0.99 ± 0.05	0.97 ± 0.09	0.094	0.98 ± 0.08	0.98 ± 0.07	0.914	0.885	0.373	0.500
Hearing	0.97 ± 0.10	0.96 ± 0.10	0.646	0.98 ± 0.07	0.97 ± 0.08	0.528	0.98 ± 0.06	0.95 ± 0.11	0.033***	0.155	0.810	0.470
Breathing	0.96 ± 0.11	0.91 ± 0.17	0.027*	0.96 ± 0.12	0.88 ± 0.18	0.007**	0.95 ± 0.12	0.90 ± 0.17	0.083	0.611	0.365	0.463
Sleeping	0.81 ± 0.21	0.80 ± 0.18	0.778	0.80 ± 0.19	0.84 ± 0.18	0.227	0.78 ± 0.22	0.83 ± 0.15	0.217	0.930	0.097	0.400
Eating	1.00 ± 0.00	1.00 ± 0.00	1.000	1.00 ± 0.00	0.99 ± 0.04	0.315	1.00 ± 0.00	0.99 ± 0.04	0.327	1.000	0.371	0.352
Speech	0.99 ± 0.04	0.98 ± 0.08	0.120	0.99 ± 0.05	0.97 ± 0.09	0.133	0.99 ± 0.05	0.97 ± 0.09	0.160	0.372	0.849	0.840
Elimination	0.66 ± 0.23	0.62 ± 0.22	0.309	0.69 ± 0.23	0.77 ± 0.23	0.069	0.72 ± 0.24	0.75 ± 0.22	0.412	0.151	< 0.001*	0.326
Usual activity	0.88 ± 0.23	0.92 ± 0.16	0.296	0.93 ± 0.20	0.93 ± 0.15	0.939	0.94 ± 0.16	0.95 ± 0.11	0.689	0.173	0.509	0.600
Mental function	0.93 ± 0.16	0.94 ± 0.13	0.433	0.95 ± 0.13	0.93 ± 0.14	0.453	0.96 ± 0.12	0.93 ± 0.14	0.223	0.372	0.699	0.445
Discomfort	0.78 ± 0.24	0.80 ± 0.25	0.598	0.87 ± 0.19	0.87 ± 0.22	0.922	0.84 ± 0.20	0.88 ± 0.20	0.210	0.013***	0.009**	0.539
Depression	0.86 ± 0.18	0.91 ± 0.14	0.098	0.89 ± 0.17	0.94 ± 0.11	0.047*	0.89 ± 0.17	0.91 ± 0.14	0.361	0.314	0.322	0.474
Distress	0.88 ± 0.19	0.92 ± 0.15	0.269	0.92 ± 0.17	0.95 ± 0.11	0.195	0.91 ± 0.17	0.93 ± 0.13	0.433	0.039***	0.204	0.364
Vitality	0.86 ± 0.18	0.87 ± 0.17	0.692	0.88 ± 0.18	0.89 ± 0.15	0.694	0.88 ± 0.20	0.88 ± 0.16	0.983	0.671	0.156	0.308
Sexual activity	0.62 ± 0.31	0.63 ± 0.34	0.851	0.82 ± 0.27	0.79 ± 0.29	0.587	0.82 ± 0.25	0.82 ± 0.28	0.990	< 0.001*	< 0.001*	0.788
15D Single Index score	0.88 ± 0.10	0.87 ± 0.09	0.472	0.90 ± 0.09	0.89 ± 0.09	0.438	0.90 ± 0.09	0.89 ± 0.11	0.460	0.005**	0.003**	0.821

Independent sample two–sided *t* test was used to compare between groups for each separate point of measure

Paired *t* test was used to compare the outcome (preoperatively, at 6 months, and at 1 year), for the two surgery groups separately

*p* value < 0.05 was considered significant

*p value < 0.001, **p value < 0.01, ***p value < 0.05

**Table 3:** The estimated EuroQol Five-Dimensional (EQ-5D) single index and sub-scores index profile values

	RASC	Uphold™		RASC	Uphold™		RASC	Uphold™		RASC	Uphold™	RASC vs Uphold™
	%, mean ± SD	Mean ± SD	*p* value	Mean ± SD	Mean ± SD	*p* value	Mean ± SD	Mean ± SD	*p* value	*p* value	*p* value	
*N*	65	69		63	62		61	62		65	69	65 vs.69
Mobility												
No problem	89.2%	80%	0.139	92.1%	87.1%	0.363	90.2%	86.9%	0.570	0.999	0.999	0.742
Slight problem/moderate	10.8%	20%		7.9%	12.9%		9.8%	13.1%				
Unable/extreme	0	0		0	0		0	0				
Self-care												
No problem	100%	98.6%	0.999	100%	100%	0.999	100%	100%	0.999	–	–	–
Slight problem/moderate	0	1.4%		0	0		0	0				
Unable/extreme	0	0%		0	0		0	0				
Usual activities												
No problem	83.1%	88.6%	0.717	92.1%	91.9%	0.493	92.2%	88.7%	0.763	0.603	0.881	0.705
Slight problem/moderate	15.4%	10%		4.8%	8.1%		8.2%	11.3%				
Unable/extreme	1.5%	1.4%		3.2%	0%		1.6%	0%				
Pain												
No problem	29.2%	36.8%	0.658	54%	69.4%	0.193	54.1%	72.6%	0.041*	0.042*	< 0.001**	0.129
Slight problem/moderate	64.6%	57.4%		41.3%	27.4%		42.6%	27.4%				
Unable/extreme	6.2%	5.9%		4.8%	3.2%		3.3%	0%				
Anxiety/depression												
No problem	60%	68.1%	0.610	68.3%	75.8%	0.382	68.9%	68.9%	0.500	0.191	0.731	0.646
Slight problem/moderate	36.9%	29%		28.6%	24.2%		27.9%	31.1%				
Unable/extreme	3.1%	2.9%	0	3.2%	0		3.3%	0				
**EQ-5D Index score**	0.85 ± 0.13	0.86 ± 0.14	614	0.89 ± 0.13	0.93 ± 0.11	0.152	0.90 ± 0.13	0.93 ± 0.1	0.093	0.024*	< 0.001**	0.359

Chi-squared and Fisher´s exact tests were used to compare groups for the nominal and categorical data respectively

ANOVA-repeated measures (preoperatively, at 6 months, and at 1 year) to compare the outcomes of the two surgery groups separately

*p* value < 0.05 was considered significant

*p value < 0.05

**p value < 0.001

**Table 4:** The quality-adjusted life-years (QALYs) as estimated from the intervention, i.e., from the hospital discharge and the follow-ups, the 15-dimensional (15D) and the EuroQol Five-Dimensional (EQ-5D) instruments

	Month start	Duration of years	RASC			Uphold™			*p* value (RASC vs Uphold™)
			*n* = 65			*n* = 71			
			Utility	Average	QALYs (mean ± SD)	Utility	Average	QALYs (mean ± SD)	
15D									
Follow-up time									
Hospital discharge	0	0.04	0.842			0.842			
2 weeks	0.46	0.21	0.866	0.854	0.032 ± 0.004 (0.031–0.033)	0.847	0.845	0.032 ± 0.004 (0.031–0.033)	0.186
3 months	3	0.25	0.901	0.884	0.187 ± 0.022 (0.182–0.193)	0.881	0.864	0.188 ± 0.024 (0.182–0.193)	0.262
6 months	6	0.25	0.904	0.903	0.225 ± 0.029 (0.217–0.232)	0.891	0.886	0.228 ± 0.030 (0.220–0.235)	0.210
9 months	9	0.25	0.901	0.903	0.221 ± 0.032 (0.214–0.229)	0.888	0.890	0.228 ± 0.028 (0.221–0.235)	0.296
12 months	12	0.25	0.903	0.902	0.220 ± 0.031 (0.213–0.228)	0.890	0.889	0.230 ± 0.024 (0.224–0.236)	0.514
QALYs (1-year)					0.898 ± 0.086 (0.845–0.921)			0.881 ± 0.082 (0.893–0.936	0.514
*p* value					< 0.001*			< 0.001*	
EQ-5D									
Follow-up time									
Hospital discharge	0	0.04	0.791			0.811			
2 weeks	0.46	0.21	0.878	0.835	0.033 ± 0.004 (0.032–0.034)	0.874	0.843	0.033 ± 0.004 (0.032–0.034)	0.775
3 months	3	0.25	0.897	0.888	0.187 ± 0.022 (0.182–0.193)	0.902	0.888	0.188 ± 0.024 (0.182–0.193)	0.950
6 months	6	0.25	0.893	0.895	0.224 ± 0.029 (0.217–0.232)	0.925	0.914	0.227 ± 0.030 (0.220–0.234)	0.598
9 months	9	0.25	0.871	0.882	0.221 ± 0.032 (0.213–0.229)	0.912	0.919	0.228 ± 0.028 (0.221–0.235)	0.213
12 months	12	0.25	0.896	0.884	0.220 ± 0.031 (0.213–0.228)	0.932	0.922	0.230 ± 0.024 (0.224–0.236)	0.046
QALYs (1-year)					0.887 ± 0.107 (0.859–0.914)			0.916 ± 0.087 (0.894–0.938)	0.295
*p* value					< 0.001*			< 0.001*	

Paired *t* test was used for statistical analysis of each cohort separately, whereas independent *t* test was used to compare the two cohorts at the measures

*p* value < 0.05 was considered significant

*p value < 0.001

**Table 5:**
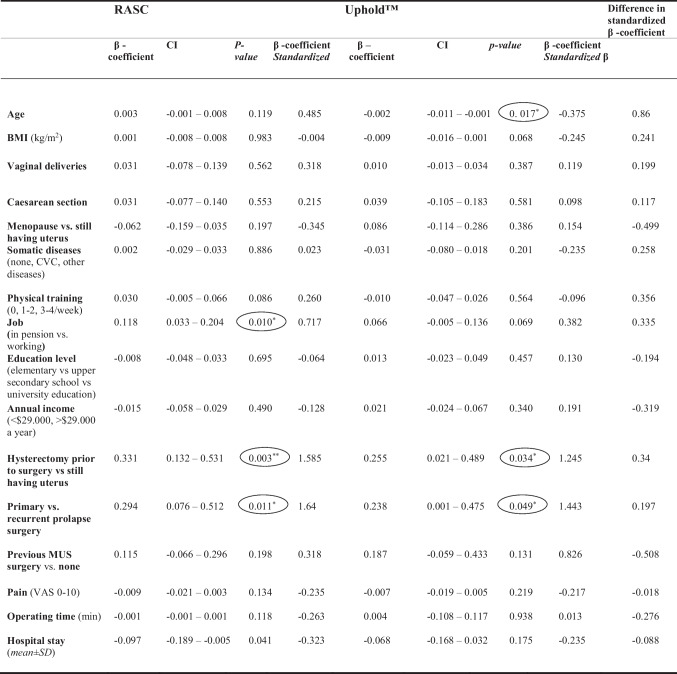
Multiple linear regression and stratified analysis of the preoperative covariates (patients´ medical and socioeconomic) and surgery characteristics effect on the 1-year quality-adjusted life-years (QALYs)

### Demographics

Patients’ medical, socioeconomic, and surgery characteristics are illustrated in detail in Table 1. There was a significant difference in age, number of vaginal deliveries, menopause, somatic diseases, previous gynecological surgery, and previous prolapse surgery in the Uphold™ group compared with the RASC group (*p* = 0.023 – *p* < 0.001). The job status was significantly higher (*p* = 0.001) and the operating time was significantly longer (*p* < 0.001) in the RASC than in the Uphold™ cohort.

### Health-Related Quality-of-Life (15-Dimensional, EuroQol Five-Dimensional Three-Level) Instruments

**Fig. 1:**
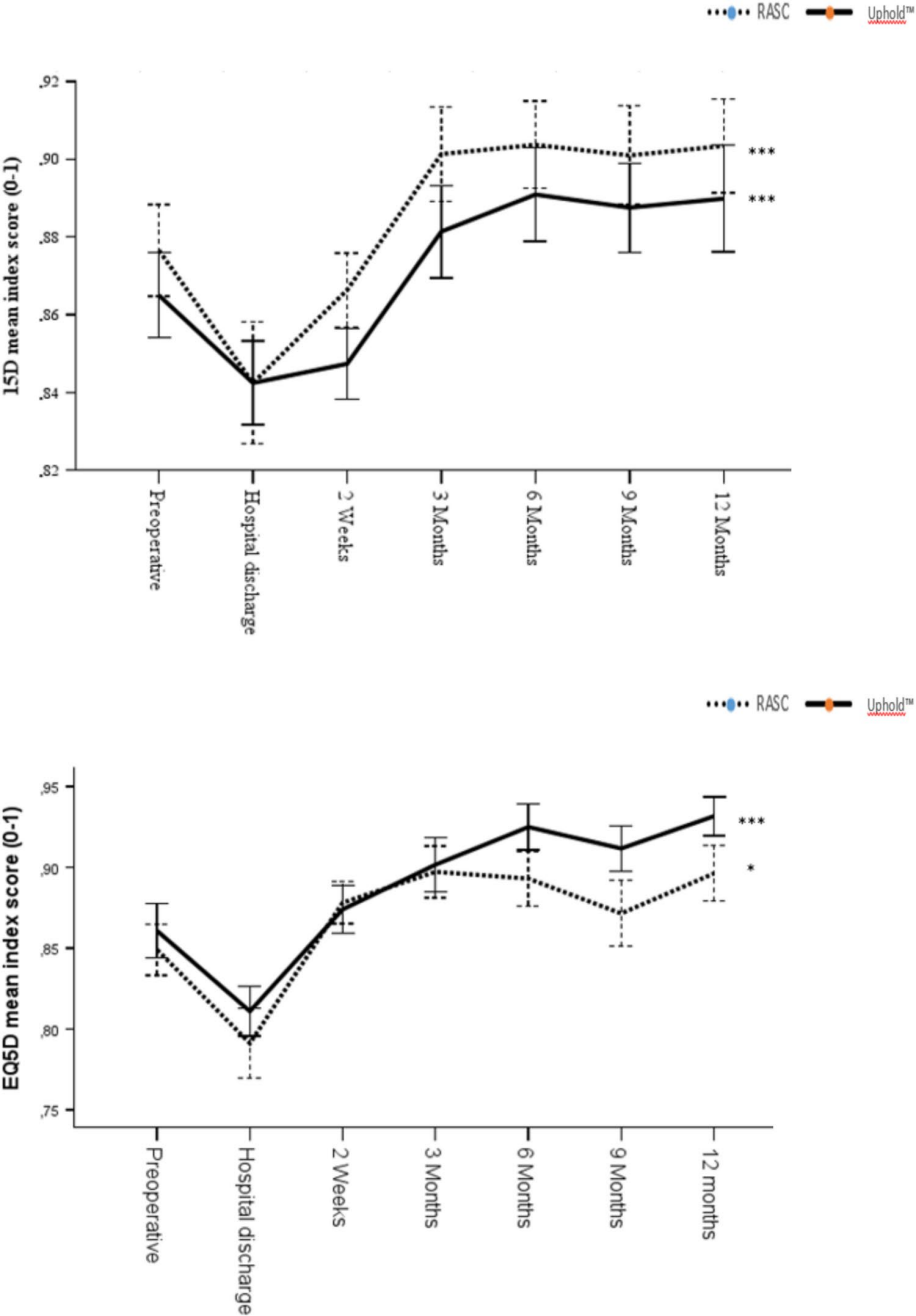
**a** 15-dimensional (15D) mean index score (preoperatively to 1 year) for the RASC versus Uphold™. **b** EuroQol Five-Dimensional (EQ-5D) mean index score (preoperatively to 1 year after surgery) for the RASC vs Uphold™

Complete information on the 15D and EQ-5D-3L instruments was provided by 65 patients in the RASC cohort whereas 71 and 69 patients in the Uphold™ cohort completed the 15D and EQ-5D-3L questionnaires respectively at the preoperative visit. Table 2 shows the estimated 15D single index score and sub-score values, whereas Table 3 shows the estimated EQ-5D-3L index score and sub-score values for the RASC and Uphold™ cohorts. Figure 1 shows changes in the 15D single index score and the EQ-5D-3L index score from preoperatively to the 1-year follow-up after surgery for the RASC and Uphold™ cohorts.

The 15D single-index score was statistically significantly improved when the preoperative 15D single-index score was compared with that 1 year after surgery (0.88 ± 0.1 to 0.90 ± 0.1; *p* = 0.005 for the RASC cohort and 0.87 ± 0.1 to 0.89 ± 0.1; *p* = 0.003 for the Uphold™ cohort, repeated measures ANOVA and paired *t* test. For the prolapse-related 15D instrument, there was a statistically significant improvement in the discomfort, distress, and sexual activity profile index scores (*p* = 0.013, *p* = 0.039, and *p* < 0.001) in the RASC cohort. For the Uphold™ cohort, there was also a statistically significant improvement in the elimination, discomfort, and sexual activity profile index scores (*p* < 0.001, *p* = 0.009, and *p* < 0.001).

**Fig. 2:**
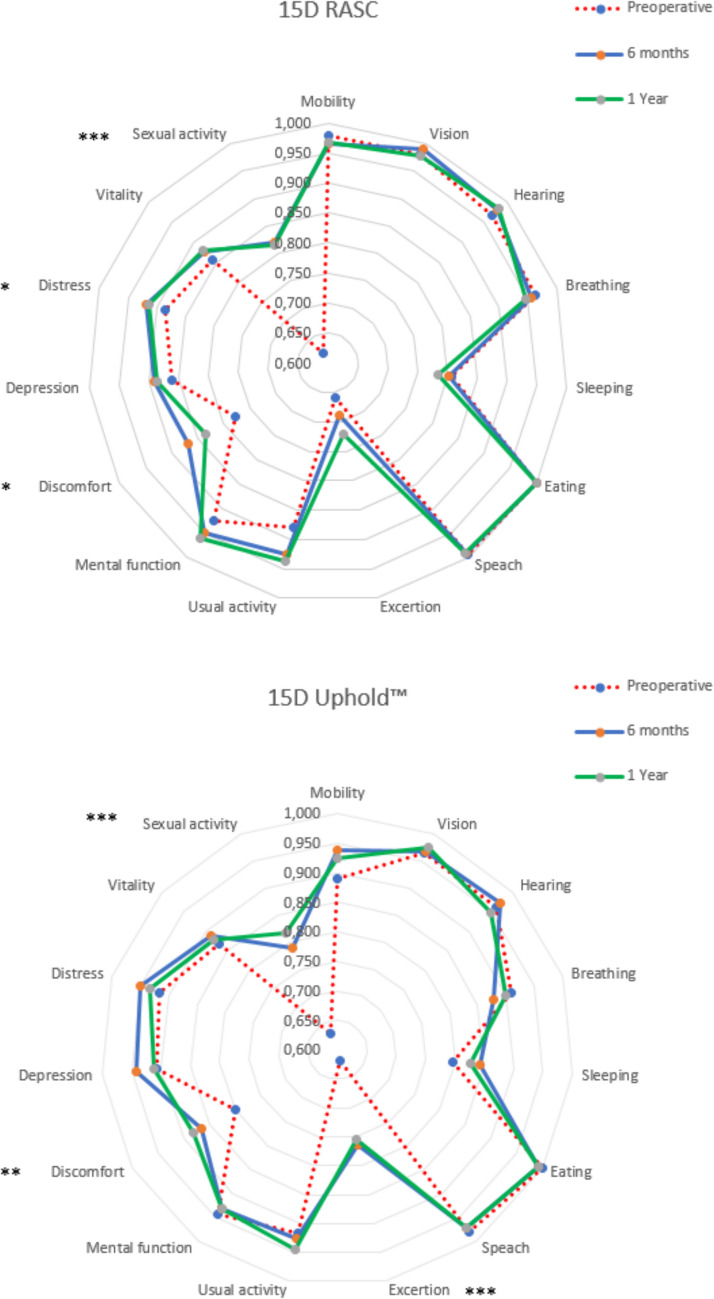
15-dimensional (15D) profile index scores (preoperatively, and 6 months and 1 year after surgery) for **a** the RASC vs **b** the Uphold™

The EQ-5D-3L index score was also statistically significantly improved at the 1-year follow-up, 0.85 ± 0.1 to 0.90 ± 0.1 (*p* = 0.009) for the RASC cohort and 0.86 ± 0.1 to 0.93 ± 0.1 (*p* < 0.001) for the Uphold™ cohort. The EQ-5D-3L pain profile index score was also statistically significantly improved at the follow-ups for the RASC and the Uphold™ cohorts (*p* = 0.024 and *p* < 0.001 respectively; Fig. 2).

### The Generic Minimal Important Change Threshold

The MIC for the 15D instrument has previously been estimated at ± 0.015 [25]. In the present study, the 15D change from the preoperative visit through 1 year after the surgery) was +0.026 for the RASC cohort and +0.025 for the Uphold™ cohort, both exceeding the 15D MIC threshold.

Also, the change in EQ-5D-3L from the preoperative through 1 year after the surgeries was +0.047 for the RASC cohort and +0.071 for the Uphold™ cohort.

### Quality-Adjusted Life-Years and Utility Evaluation

The estimated QALY gain and the utility are shown in Table 4. Using the 15D instrument, the evaluated 1-year QALYs was estimated to be 0.90 ± 0.1 and 0.88 ± 0.1 for the RASC and the Uphold™ cohorts respectively (*p* < 0.001). Similar results were noticed when using the EQ-5D-3L for estimating the QALYs, 0.89 ± 0.1 and 0.92 ± 0.1 for the RASC group and the Uphold™ group respectively (*p* < 0.001). The estimated QALY values of the RASC and Uphold™ cohorts were comparable, and no statistical significance was found between the surgery cohorts for the estimated QALYs at any of the follow-up visit, when using the 15D or the EQ-5D-3L instruments.

The 15D utility at the 1-year follow-up was estimated to be 0.90 (average 0.9) for the RASC cohort and 0.89 (average 0.89) for the Uphold™ cohort (Table 4). Similar results were also noticed when using the EQ-5D-3L, i.e., the utility was valued at 0.89 (average 0.88) for the RASC cohort and 0.93 (average 0.92) for the Uphold™ cohort (Table 4).

### Effects of Patient Medical, Socioeconomic, and Surgery Characteristics on the 1-Year Quality-Adjusted Life-Years Gained

There was a statistically significant difference in patient medical, socioeconomic, and surgery characteristics between the cohorts, which may be confounders. For this reason, we used a multiple linear regression model to adjust for the confounder effect on the QALYs. All results from the multiple linear regression analysis investigating the impact of patients’ medical, socioeconomic, and surgical characteristics on the estimated QALYs are shown in Table 5. Job status was associated with an improvement in QALYs gained for the RASC cohort (β-coefficient 0.118, CI −0.033 to 0.024 and *p* = 0.010), i.e., correlated with QALY improvements. For the Uphold™ cohort, age had a small negative correlation with the QALY gain (β-coefficient −0.002, CI −0.011 to −0.001 and *p* = 0.017) indicating that older patients gained fewer QALYs. Hysterectomy prior to the primary surgery had a large positive association, with QALYs gained in both the RASC and the Uphold™ cohorts (β-coefficient 0.331, CI 0.132 to 0.531, and *p* = 0.003, and β-coefficient 0.255, CI 0.021 to 0.489, and *p* = 0.034 respectively). A similar correlation was also observed for prolapse recurrence before the primary surgery by the RASC and the Uphold™ cohorts with a positive association with the QALYs gained (β-coefficient 0.294, CI 0.076–0.512, and *p* = 0.011, and β-coefficient 0.238, CI 0.001–0.475, and *p* = 0.049 respectively). No other demographic, socioeconomic, and surgery characteristics had a statistically significant influence on the QALYs gained.

## Discussion

In this secondary analysis of women undergoing minimally invasive abdominal mesh versus anterior–apical vaginal mesh repair of symptomatic apical prolapse, there was a similar and significant improvement in quality-of-life indexes as estimated by the 15D and EQ-5D-3L HR-QoL instruments. There was a significant improvement in the prolapse-related 15D profile index scores in the two cohorts. The MIC threshold was achieved in both cohorts, which indicates that both the RASC and the Uphold™ are clinically meaningful surgical treatments for prolapse. The estimated QALYs gained indicates a good state of health 1 year following both surgical interventions. The HR-QoL improvement for the two surgery cohorts exceeded the improvement threshold for the MIC and therefore reflects a clinically important improvement. Furthermore, there was no statistically significant difference in the HRQoL improvement between the cohorts. The results are compatible, with RASC and the Uphold™ representing comparable and equally meaningful surgeries for prolapse repair, with the improved HR-QoL and the QALYs gained 1 year after surgery.

The SCP has been considered to be the criterion standard procedure for pelvic organ prolapse repair and to be superior to all vaginal surgery procedures [1]. A growing body of evidence indicates that the Uphold™ is efficacious in restoring the prolapse anatomy in the short and long term [7–11]. A recent randomized controlled study indicates that the composite primary efficacy of the Uphold™ is non-inferior to the sacrocolpopexy [12]. Furthermore, the reoperation rate for prolapse recurrence within 1 year after surgery was lower in the Uphold™ group than in the RASC group [13]. The majority of previous studies focused on estimating the local symptoms, i.e., the disease-specific subjective pelvic floor outcome. However, the generic quality-of-life and health-economic aspects have been understudied and there is a lack of knowledge when comparing how the RASC and the Uphold™ may have affected the HR-QoL and health-economic gains.

The 15D and EQ-5D-3L instruments have been widely used to investigate the generic HR-QoL in several medical specialties, but they have been scarcely used in pelvic floor surgery [16, 17]. An inverse correlation has been shown between the improved generic HR-QoL, as estimated by the 15D instrument, and the decrease in the subjective disease-specific pelvic floor outcomes after prolapse surgery using the Uphold™ [16]. In the present study, we found a significant improvement in the generic HR-QoL, as estimated by the 15D and the EQ-5D-3L instruments 1 year after surgery in the RASC and Uphold™ cohorts. Improvement in generic HR-QoL in both cohorts was correlated to statistically significant improvements in the 15D prolapse-related profile index scores. The estimated 15D and EQ-5D-3L improvements exceeded typical MIC in the RASC and the Uphold™ cohorts. These results indicate improvement in the generic HR-QoL in the two cohorts, with no significant difference between them.

The 1-year QALY gain was calculated using 15D equal to 0.90 ± 0.1 for RASC and 0.88 ± 0.1 for the Uphold™ (*p* < 0.001). Similar results were confirmed when using the EQ-5D-3L to evaluate the 1-year QALYs, 0.89 ± 0.1 for the RASC, and 0.92 ± 0.1 for the Uphold™ (*p* < 0.001). These results indicate that the RASC and the Uphold™ are meaningful surgical treatment methods for apical prolapse. Previous studies in urogynecological surgery indicated that several factors, including smoking, previous hysterectomy, age, and BMI had influenced the QoL-related outcomes [26–28]. For this reason, and in order to minimize the bias effect, we performed a multiple linear regression and stratified analysis. From the present study, previous hysterectomy and previous prolapse surgery before RASC and the Uphold™ had a positive effect on the QALYs gained. Furthermore, education level had a positive association, with QALYs gained in the RASC cohort. Conversely, age had a negative association with QALYs gained in the Uphold™ cohort.

Among the strengths of the present study are the use of validated HR-QoL outcome measures, the multicenter setting, the high-volume surgery centers, the standardized surgical methods, and the prospective and controlled study form. The satisfactory compliance during follow-up strengthens our results by minimizing selection and reporting bias.

The present study has several important limitations. A major limitation is the intrinsic selection bias of surgeons in choosing the abdominal or the vaginal mesh surgeries. Although we adjusted for the differences in demographic characteristics using a logistic regression analysis, a bias may remain a potential confounder. We may recognize that an independent control group, i.e., patients with prolapse undergoing native tissue repair without synthetic mesh, would have added valuable information to understanding of the advantage of surgery types regarding HR-QoL and QALYs. The reoperation rate within 1 year was higher in the RASC cohort but was not investigated in the present study when calculating the QALY gain. However, the reoperation rate alone or in combination with other factors, including surgery cost, may affect the cost effectiveness. A longer follow-up may also provide important information on the health-economic gains. It is beyond the present study to compare the cost-effectiveness analysis of the two surgery cohorts. Future studies should assess cost effectiveness to provide evidence for health-technology assessors to optimize societal resource allocation in apical prolapse surgery settings and more broadly across entire health care budgets.

In summary, we found improvement in the generic HR-QoL and QALY gain 1-year after prolapse surgery using RASC and Uphold™ vaginal mesh. These results were confirmed by two different HR-QoL instruments, the 15D and the EQ-5D-3L. The improvement in the HR-QoL and the QALYs gained indicates that the RASC and the Uphold™ are meaningful surgical treatments for prolapse.
